# Owners’ Experience and Satisfaction with Radioiodine Treatment in Hyperthyroid Cats—A Prospective Questionnaire Study

**DOI:** 10.3390/vetsci12050458

**Published:** 2025-05-10

**Authors:** Sofie Muthmann, Joana Léonie Tiefenbrunner, Fabienne Blunschi, Isabell Klemm, Natali Bettina Bauer, Katarina Hazuchova

**Affiliations:** Clinic for Small Animals (Internal Medicine, Clinical Pathology and Clinical Pathophysiology), Justus-Liebig University Giessen, 35392 Giessen, Germany; joana.tiefenbrunner@vetmed.uni-giessen.de (J.L.T.); fabienne.blunschi@vetmed.uni-giessen.de (F.B.); isabell.mueller@vetmed.uni-giessen.de (I.K.); natali.bauer@vetmed.uni-giessen.de (N.B.B.); katarina.hazuchova@vetmed.uni-giessen.de (K.H.)

**Keywords:** hyperthyroidism, quality of life, radioiodine treatment, owner motivation

## Abstract

Radioiodine (RAI) treatment (RAIT) is considered the gold standard for treatment of feline hyperthyroidism. This study aimed to assess owners’ motivation, concerns and satisfaction with RAIT and health-related quality of life (HRQoL) changes in RAI-treated cats. Two surveys (before and six months post-RAIT) were sent to owners of cats scheduled for RAIT between April 2023 and March 2024. The owners of 78 and 68 cats completed the first and the second surveys, respectively. The main reasons for choosing RAIT were that RAIT was considered the gold standard treatment (*n* = 27/78; 35%) and difficulties administering antithyroid drugs (*n* = 18/78; 23%). The primary care veterinarian (*n* = 50/78; 64%) and the internet (*n* = 33/78; 42%) were the main information sources about RAIT at the referral clinic. Owners were mostly concerned about the anaesthetic risk and hospitalisation, with the cat missing the owner and vice versa being the main worries. Most owners were satisfied with the outcome and their decision for RAIT. The HRQoL score improved within the first six months after RAIT (*p* < 0.01), with no difference between euthyroid and hypothyroid cats (*p* = 0.609). This study emphasises the role of the primary care veterinarian and the internet as primary sources of information regarding RAIT. The findings help to better understand owner concerns, improve owner counselling, and educate primary care veterinarians about RAIT.

## Simple Summary

Radioiodine treatment is considered the gold standard treatment for hyperthyroidism in cats. However, little research exists addressing owner concerns and experiences with radioiodine treatment. This study explores why owners choose radioiodine treatment, their worries and satisfaction, and changes in their perception of their cat’s health-related quality of life after treatment. Cat owners were asked to complete two online surveys (one before and one six months after radioiodine treatment). A total of 78 owners completed the first survey and 68 completed the second survey. The main reasons for choosing radioiodine treatment were its status as the “gold standard” (35%) and difficulty giving antithyroid medication (23%). Veterinarians (64%) and the internet (42%) were the most common information sources. The biggest concerns were anaesthesia risks and separation from their cats during hospitalisation. After treatment, most owners were satisfied with their decision for and the outcome of radioiodine treatment. The health-related quality of life improved significantly, and there was no difference between cats that had normal (euthyroid) or low thyroid values (hypothyroid). This study highlights the important role of primary care veterinarians and online resources in decision-making processes and provides valuable insights to improve owner counselling and support and education of primary care veterinarians.

## 1. Introduction

Hyperthyroidism is the most common endocrine disease in middle-aged to older cats [1]. Radioiodine treatment (RAIT) is considered the gold standard for managing this disease, as it targets all abnormal thyroid tissue, is minimally invasive and is usually curative (>95% of the cases) [2,3,4]. However, in comparison, fewer owners choose RAIT than antithyroid drugs (ATDs) [5,6,7]. One of the reasons for this treatment choice might be the lack of awareness. In two previous studies, 29.7% and 50% of respondents, respectively, reported that they had no knowledge of RAIT, as they were not informed about this option by their primary veterinarian [8,9]. Furthermore, factors such as the advanced age of the cat (>15 years), lack of insurance and the relatively high costs of RAIT might be reasons why owners decide against RAIT [8,10].

Owners who choose RAIT often do so to improve their cat’s health. The most common reasons for choosing RAIT are difficulties administering ATDs, adverse effects of previous therapies [10], or personal belief that RAIT is the best treatment option [8]. Despite initial concerns, most owners reported high satisfaction with RAIT [8,10] alongside an improvement in their cat’s quality of life (QoL) following RAIT [8]. However, this QoL assessment was based on a simple rating scale [8] rather than using a validated QoL tool, which might fail to capture the multidimensional nature of QoL [11].

While previous studies provided some important insights into owners’ perceptions regarding RAIT, they were retrospective, and in some cats several years have passed between the RAIT and completion of the questionnaire, introducing potential recall bias and fading affect bias [12,13]. Prospective studies evaluating owners’ expectations and concerns immediately before and a short time after RAIT but leaving sufficient time for the normalisation/stabilisation of thyroid function [5] would be useful to reduce potential biases. The implementation of a validated QoL questionnaire, such as the recently published Hyperthyroidism Cat-QoL [5], would allow for an objective evaluation of changes in QoL after RAIT.

The aim of this study was to find out more about why owners choose RAIT, how they experience the hospitalisation following RAIT, which aspects of the treatment they find most challenging, and their satisfaction with RAIT. The second aim of this study was to assess the cats’ QoL as reported by the owner before and six months after RAIT using the validated Hyperthyroidism Cat-QoL [5] to objectively determine the extent of the QoL improvement, if present, and whether the improvement is associated with the outcome.

## 2. Materials and Methods

### 2.1. Study Design

This was a prospective, questionnaire-based study. All owners who had an appointment for RAIT at the Clinic for Small Animals of the Justus-Liebig-University Giessen, between April 2023 and March 2024, were contacted by email one week before the appointment and offered participation in the study aiming to assess their opinions regarding RAIT and QoL of their cats. Owners who consented to participate were asked to complete two sets of questionnaires, the first within a week of the RAIT and the second six months following RAIT. Besides questions concerning RAIT and assessing the health-related QoL (HRQoL) of the cat using the Hyperthyroidism QoL-cat [5], the first set of questionnaires (prior to RAIT) also asked for information about the cat and previous hyperthyroidism treatment and sociodemographic information about the owner. The second set of questionnaires (six months post-RAIT) included only questions concerning RAIT and assessment of the cat’s HRQoL.

All cats presented for RAIT within the study period whose owners consented to participate were included in this study and, concurrently, in a study investigating the effect of owner personality and different hyperthyroidism treatment modalities (including RAIT) on HRQoL of hyperthyroid cats [14]. The only exclusion criterion was the submission of an incomplete questionnaire. Questionnaires from owners, who only completed the first questionnaire but not the second (six months later) (e.g., because the cat died), were still included in the analysis of the initial questionnaire. The questionnaires were made available online in German and English using an online survey program (LimeSurvey GmbH, Hamburg, Germany).

#### 2.1.1. Standard Procedures Before, During, and After RAIT at the Clinic for Small Animals and Radiation Safety Measures for Owners

In all cats referred for RAIT to the Clinic for Small Animals, all available medical records of the cats are reviewed by a team of veterinarians with special interest in hyperthyroidism and RAIT prior to scheduling the appointment, and any owner questions regarding RAIT are answered via email or telephone. The owners are advised to stop ATDs 7 days and a low iodine diet (LID) 14 days prior to RAIT. All cats are admitted to the hospital two days before RAIT in order to carry out the necessary pre-treatment tests (including haematology, clinical chemistry, total thyroxine [TT4] and thyroid stimulating hormone [TSH] measurement, urinalysis, thoracic radiographs and blood pressure measurement) to ensure that cats are stable enough to undergo sedation for scintigraphy and hospitalisation in isolation and allow the cats to get used to the hospital environment. Two days after admission, scintigraphy followed by RAIT is performed under sedation. Following RAIT, cats are hospitalised in isolation in a specialised radiation safety ward, where handling of the cats is restricted, and strict radiation safety protocols are followed. During hospitalisation, the owners receive daily phone call updates from the attending clinician. Residual radiation levels are usually assessed on the fourth and fifth days post-treatment to determine the appropriate time for discharge from the hospital. Depending on the administered iodine dose, the cats are usually discharged after five to ten days following RAIT (rarely, cats receiving a high dose of iodine of up to 30 mCi for the treatment of a suspected thyroid carcinoma [15] might need to be hospitalised for longer time periods). Upon discharge, owners are instructed to adhere to strict radiation safety measures at home. Direct contact with the cat should be limited to no more than two hours per day, cat litter containing the excrements must be stored separately, and gloves must be worn during litter box cleaning. Additionally, the cats must remain indoors for four weeks and contact with pregnant women and young children must be avoided.

#### 2.1.2. Hyperthyroidism Cat-QoL

The QoL of the cats was assessed both before and six months after RAIT using the validated Hyperthyroidism QoL-cat questionnaire [5]. This questionnaire has been validated in both English and German and consists of 25 items distributed across four domains: three cat-related domains (19 items: ‘gastrointestinal, dietary, urination’ [7 items], ‘appearance’ [3 items], and ‘activity and behaviour’ [9 items]), one owner-related domain (6 items), and one item to rate the cat’s QoL on a scale from 1 to 5. Each of the 25 items includes two questions that assess the quantity and the quality of the effect each item has on the cat’s QoL. The questions are rated on a scale from 0 to 4. The item score is calculated by multiplying the scores of the two questions, and the item scores are summed to calculate the total HRQoL score, ranging from 0 (best possible HRQoL) to 382 (worst possible HRQoL) [5]. When completing the HyperthyroidismCat-QoL, the owners were instructed to refer their answers to the past four weeks, prior to the discontinuation of any ATDs or LID treatment.

#### 2.1.3. Questions Prior to RAIT

The first questionnaire (to be completed before the RAIT) consisted of 62 questions (including 26 questions concerning the HRQoL assessment). The wording of the questions can be found in the Supplementary Materials. A link was sent to the owners by email one week prior to their RAIT appointment, and they were reminded two to three times.

General data about the cat and its hyperthyroidism treatment (signalment, duration of hyperthyroidism and severity of clinical signs, previous hyperthyroidism treatment, current level of hyperthyroidism control [i.e., thyroid status: eu-, hypo- or hyperthyroid], comorbidities and their treatment, etc.) were collected. The thyroid status prior to RAIT (before discontinuation of ATDs or an LID as described in Section 2.1.1) and any comorbidities and their treatment were recorded as stated by the owners but were not confirmed by inspection of medical records. The owners were asked to rate their concern about their cat’s hyperthyroidism on a scale ranging from 1 (not a concern at all) to 10 (extremely concerned). Furthermore, information about the sociodemographic background of the owner (e.g., gender, age, highest achieved level of education, etc.), the time the owner spent with their cat, whether they were the primary carer of the cat, and whether this was their first cat and how many cats they currently had was also obtained. These questions mainly required a “tick-box” answer.

The owners were also asked eight questions focusing on their decision-making process and concerns regarding the RAIT. The first five questions required a “tick-box” answer and addressed topics such as why the owners chose RAIT, how they learned about this treatment option, the travel distance to the treatment facility, the waiting period for the RAIT appointment, and whether they had insurance covering the costs of RAIT. In addition, three questions rating different statements regarding owners’ concerns related to RAIT, hospitalisation and satisfaction with the information provided by the clinic prior to RAIT on a scale ranging from 1 (not a concern at all/not satisfied at all) to 10 (extremely concerned/extremely satisfied) were included.

#### 2.1.4. Questions Six Months After RAIT

The second set of questionnaires (six months post-RAIT) consisted of 46 questions (including 26 questions concerning the HRQoL assessment); the wording of these questions can be found in the Supplementary Materials. A link was sent to the owners by email, and they were reminded twice at intervals of about two to three weeks.

Owners were asked questions about the severity of their cat’s clinical signs and when they first observed significant improvement of their cat’s symptoms, the thyroid status of their cat, and any comorbidities diagnosed since RAIT and whether these required any treatment. Additionally, owners were asked to rate their level of concern about their cat’s hyperthyroidism on a scale ranging from 1 (not a concern at all) to 10 (extremely concerned).

Owners were also asked to rate the following aspects on a scale ranging from 1 to 10: their satisfaction with the information provided prior to RAIT; their experience during hospitalisation; any difficulties with reintegration of their cat following discharge from the hospital; the burden associated with abiding by the radiation safety measures; their opinion on re-examinations following RAIT; their satisfaction with their decision for their cat to undergo RAIT and with the outcome of RAIT; and what they perceived as the advantages and disadvantages of RAIT. Finally, owners were asked whether they would recommend RAIT to others (single choice).

#### 2.1.5. Evaluation of Treatment Outcome 6 Months Post-RAIT

Treatment outcome was defined as resolution of hyperthyroidism in cats with at least one TT4 measurement within or below reference interval (RI) following RAIT or treatment failure in cats with persistent hyperthyroidism (TT4 > RI six months following RAIT) [16]. In cats that participated in the re-examinations offered by the Clinic for Small Animals, which were performed at four weeks, three months, and six months after RAIT in agreement with current literature [16,17], thyroid status six months after RAIT could be assessed more precisely based on TT4 and TSH measurement. If available, the treatment outcome was therefore based on the clinical records of the cats. Based on these results, cats were classified as euthyroid, hyperthyroid, subclinical hypothyroid, or overt hypothyroid. The classes were defined as follows: euthyroid: TT4: 1–4 µg/dL [12.9–51.5 nmol/L] and TSH < 0.44 µU/mL [<3.19 pmol/L]), hyperthyroid (TT4 > 4 µg/dL [>51.5 nmol/L]), or hypothyroid (subclinical hypothyroidism: TT4 1–2.5 µg/dL [12.9–32.2 nmol/L] and TSH > 0.44 µU/mL [>3.19 pmol/L]; overt hypothyroidism: TT4 < 1 µg/dL [<12.9 nmol/L] and TSH > 0.44 µU/mL [>3.19 pmol/L]) [16]. Hypothyroid cats with new onset of azotaemia (creatinine ≥ 140 µmol/L) were treated with levothyroxine (LT4). Levothyroxine was started at a dose of 100 µg/cat once daily, which was increased monthly by 50 µg until a TT4 concentration of 2.5–3.5 µg/dL was achieved [16].

### 2.2. Statistical Analysis

Data were downloaded from Lime Survey as Excel files (Microsoft Corp., Redmond, WA, USA). Excel was also used for descriptive analysis and to calculate the HRQoL scores. SPSS (IBM SPSS, version 26, IBM Germany GmbH, Schorndorf, Germany) was used for statistical analysis. Data were assessed for normality using the Shapiro–Wilk test and visual analysis of histograms. Because most data were non-normally distributed, they are presented as medians (interquartile ranges [IQR]). Categorical data are presented as numbers and percentages (proportions). The information obtained from the first set of questionnaires (prior to RAIT) is presented for all participating cats/owners. For a subset of cats, whose owners completed the second set of questionnaires six months following RAIT, the HRQoL score and the severity of clinical signs prior to and six months after RAIT were compared using the Wilcoxon signed rank test. The Mann–Whitney U test was used to compare the HRQoL of hypothyroid and euthyroid cats (only cases with available TT4 and TSH measurement) six months after RAIT. Statistical significance was set at *p* < 0.05. Bonferroni correction was applied to adjust *p*-values for multiple comparisons.

## 3. Results

**Figure 1:**
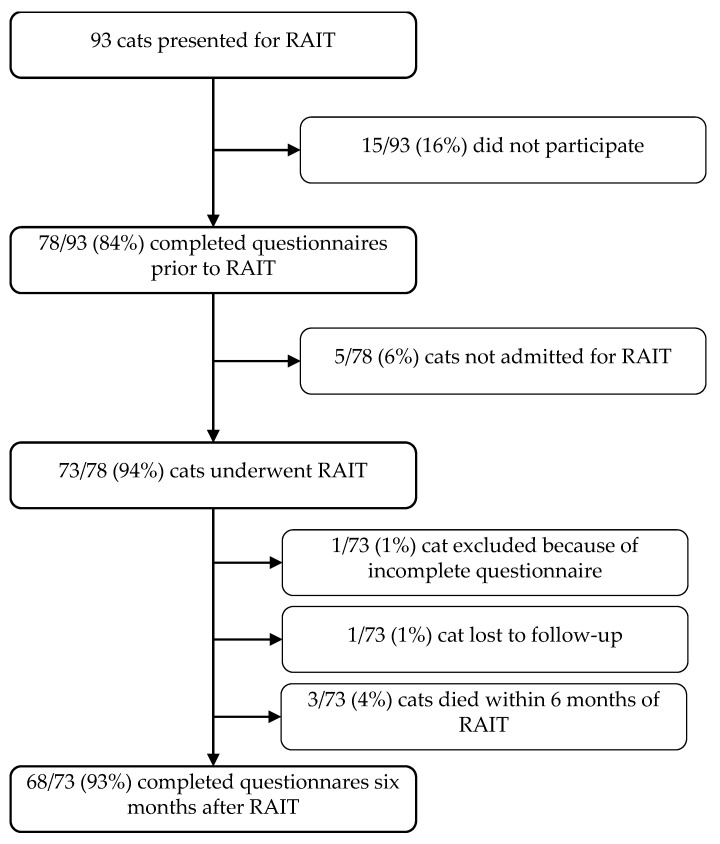
Flowchart describing the study population before and six months after radioiodine treatment (RAIT).

The study population is presented in Figure 1. A total of 93 cats presented for RAIT during the study period. The owners of 15 (*n* = 15/93, 16%) cats did not participate for unknown reasons; therefore, the first set of questionnaires was completed for 78 cats (*n* = 78/93; 84%). Of these 78 cats, 5/78 (6%) could not undergo RAIT because of serious cardiac or respiratory disease, 3/78 (4%) cats died within six months of RAIT, 1 cat (1/78, 1%) was lost to follow-up, and the 6-month questionnaire of 1 cat (1/78, 1%) was incomplete and had to be excluded. Therefore, the second set of questionnaires (six months post-RAIT) was available for 68/78 (93%) cats.

### 3.1. Characteristics of Owners of RAI-Treated Cats

Because one owner presented for RAIT with two cats, information on 77 owners is described below. All 77 owners lived in Germany, but five owners (*n* = 5/77; 7%) completed the questionnaire in English. The majority of participants were female (*n* = 60/77; 78%) and over 50 years old (*n* = 31/77; 40%). Seven owners (*n* = 7/77; 9%) had children under the age of 18. Almost half of the owners (*n* = 35/77; 46%) worked full time, and the highest educational qualification was a completed vocational training (*n* = 21/77; 27%), followed by a master’s degree (*n* = 12/77; 16%). A full list of the sociodemographic data of the owners can be found in the Supplementary Materials.

Most participants stated that they were the main carer of the cat (*n* = 64/77; 83%). For about one fifth of the owners (*n* = 17/77; 22%), this was their first cat and seven owners (*n* = 7/77; 9%) had previously had a cat with hyperthyroidism. Most owners owned one (*n* = 30/77; 39%) or two cats (n = 33/77; 43%), but almost a fifth had three or more cats (n = 14/77; 19%). Just over half of the owners (*n* = 44/77; 56%) spend more than seven hours a day with their cat; the others spend less than seven hours (*n* = 33/77; 44%).

### 3.2. Characteristics of the Cats Presented for RAIT

Of the 78 included cats, the majority were male (53%), aged between 11 and 14 years old (64%), and of the European Shorthair breed (71%) (Table 1). Further details about the signalment are presented in Table 1.

Half of the cats (*n* = 40/78, 51%) were strictly indoor; the remaining cats were outdoor but came into the house regularly (*n* = 18/78; 23%); were allowed outdoors in the garden but only under supervision (*n* = 16/78; 21%); or were allowed outdoors but only on a leash (*n* = 4/78; 5%).

Information about the cat’s duration of hyperthyroidism, treatment of hyperthyroidism prior to presentation for RAIT, last known thyroid status prior to RAIT, frequency of thyroid hormone measurements, comorbidities and their treatment is provided in Table 2. Almost half of the cats had been hyperthyroid for less than six months (42%). Most cats were treated with ATDs prior to the decision to undergo RAIT (73%). The last known thyroid status prior to the RAIT appointment, as stated by the owner, was hyperthyroid or euthyroid for 44% of the cats each. Around a third of the owners (29%) had their cat’s thyroid hormone concentrations checked at the veterinarian four times a year before RAIT. According to their owners, almost half of the cats (47%) had no other known comorbidity. Among cats with comorbidities, dental disease (35%) was the most common. Most cats did not receive any additional treatment (73%) other than treatment for hyperthyroidism.

### 3.3. General Data Relating to the Decision of the Owner in Favour of a RAIT

Information about why the owners chose RAIT for their cat, how they obtained information about RAIT, how long they waited for the appointment and how far they travelled to our hospital is summarised in Table 3. For about a third of the cats (35%), owners chose RAIT because it is the gold standard treatment for hyperthyroidism; side effects of the ATDs were the second most common reason for choosing RAIT (23%). The owners of most cats (64%) were informed about the possibility of RAIT at our hospital by their primary care veterinarian or found the information on the Internet (42%). The travel distance for most of the owners and cats was below 300 km, and the vast majority (90%) waited two months or less for the appointment.

Only three cats (*n* = 3/78; 4%) had health insurance that covered the costs of the RAIT. The owners of most cats were very satisfied with the information provided by the clinic prior to RAIT (median: 9; IQR: 8–10; 10 is consistent with the highest degree of satisfaction).

### 3.4. Concerns About RAIT and Hospitalisation Expressed by the Owners Prior to RAIT

**Figure 2:**
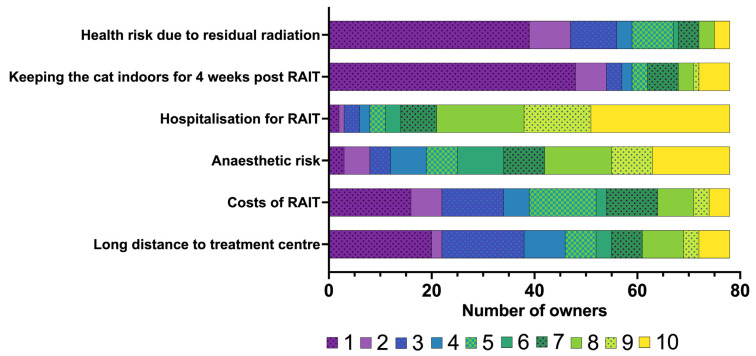
Bar chart summarising the concerns of the owners of the 78 cats included in the study regarding radioiodine treatment (RAIT) prior to RAIT, rated on a scale from 1 to 10 (1 = not a concern for me at all to 10 = concerns me strongly). The different numbers on the scale from 1 to 10 assigned by the owners are colour-coded (the legend for the colour scheme is within the figure).

**Figure 3:**
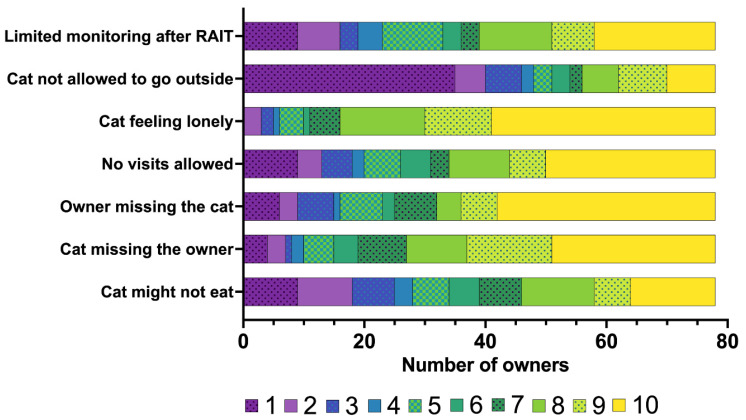
Bar chart summarising the concerns of the owners (*n* = 78) regarding the hospitalisation of their cat after radioiodine treatment (RAIT), rated on a scale from 1 to 10 (1 = not a concern for me at all to 10 = concerns me strongly). The different numbers on the scale from 1 to 10 assigned by the owners are colour-coded (the legend for the colour scheme is within the figure).

The owners’ rating of their concerns prior to RAIT is summarised in Figure 2, with concerns specifically related to hospitalisation following RAIT presented in Figure 3.

Neither the long journey (median: 4; IQR: 1.25–7) nor the costs associated with RAIT (median: 4.5; IQR: 2–7) were of major concern for owners who have chosen RAIT (Figure 2). Keeping the cat indoors for an additional 4 weeks (median: 1; IQR: 1–4) or the health risk to the owner due to residual radiation (median: 1.5; IQR: 1–4) was also not a major worry (Figure 2). The owners were most concerned about hospitalisation after RAIT (median: 9; IQR: 7–10) and about the anaesthetic risk (median: 7; IQR: 5–9) (Figure 2). Most owners felt that their cat might feel lonely (median: 9; IQR: 8–10), that they themselves would miss their cat (median: 9; IQR: 5–10) and that the cat would miss its owner very much during the hospitalisation after RAIT (median: 9; IQR: 7–10) (Figure 3). The owners were also worried that only limited monitoring after RAIT is possible (median: 7.5; IQR: 4–9.75), that the cats could not be visited (median: 8; IQR: 8–10), and might not eat (median: 6.5; IQR: 3–8.75), but they were less worried about the need to stay indoors during the hospitalisation (median: 2; IQR: 1–8) (Figure 3).

### 3.5. Outcome of the RAIT and Comparison of Clinical Signs and QoL Before and Six Months After RAIT

Of the 68 study cats whose owners completed the questionnaire six months post-RAIT, the clinical records of 58 cats (85%) were available to define the outcome of RAIT.

Hyperthyroidism resolved in 56 cats (*n* = 56/58, 97%), but 2 cats (*n* = 2/58, 3%) remained persistently hyperthyroid. Of the 56 cats with resolution of hyperthyroidism, 31 cats (*n* = 31/56, 55%) became euthyroid, and 25 (*n* = 25/56, 44%) became hypothyroid (with 21/25 [84%] requiring LT4 supplementation). In 10 cats (*n* = 10/68, 15%), the laboratory results of the 6-month re-examination were not available, as the owners (*n* = 9/10) had chosen to have the check-up conducted by their primary veterinarian. One owner (1/10) did not conduct the re-examinations at all. As a result, it was not possible to reliably differentiate between eu- and hypothyroidism in these ten cases. According to the owners, 8/10 cats were euthyroid and 1/10 was hypothyroid six months after RAIT.

There was a significant improvement in all clinical signs between enrolment and month 6 following RAIT (Table 4). In most cats, clinical signs of hyperthyroidism improved for the owners within the first month (*n* = 26/68, 38%) or one to two months (*n* = 26/68, 38%) following RAIT. In only a minority of the cats (*n* = 9/68, 13%), it took three to six months for the clinical signs to improve, and five owners reported no improvement (*n* = 5/68, 7%).

**Figure 4:**
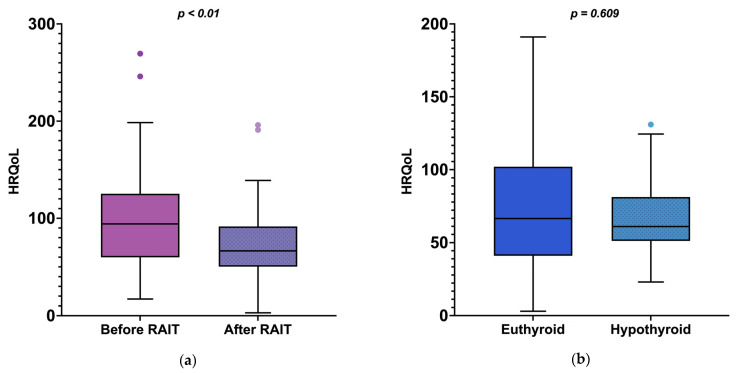
Box-whisker plots representing health-related quality of life (HRQoL) scores: (**a**) before and six months after radioiodine treatment (RAIT) in 68 cats, and (**b**) in confirmed euthyroid (*n* = 31/68; 46%) and confirmed hypothyroid (*n* = 25/68; 37%) cats. Low HRQoL scores reflect a better, and higher scores a worse, quality of life. Boxes represent the interquartile range (IQR); the central line within the box shows the median value, the whiskers denote the range extending out to 1.5x IQR, and data points that were >1.5 IQR are shown as circles.

Health-related QoL also significantly improved between enrolment and month 6 (*p* < 0.01, Table 4, Figure 4a). Importantly, for cats with resolution of hyperthyroidism where precise information regarding thyroid status was available at month 6 (*n* = 56), there was no difference in HRQoL between cats that became euthyroid (*n* = 31) and those that became hypothyroid (*n* = 25) following RAIT (*p* = 0.609, Figure 4b). The owners’ general concern about hyperthyroidism also significantly improved at month 6 when compared to enrolment (*p* < 0.01, Table 4). Few cats developed comorbidities, which, together with their treatment, are listed in Table 5.

### 3.6. Owners’ Perception of the Hospitalisation and the Immediate Period Following Discharge from the Hospital, Including Radiation Safety Measures

Similarly to their opinion prior to admission for RAIT (presented in Section 3.3), six months later, the owners were still satisfied with the information and support they obtained from our clinicians prior to treatment (median: 10; IQR: 8.8–10; 10 representing the highest degree of satisfaction). The satisfaction with information provided by the attending clinicians during the hospitalisation was also high (median: 10; IQR: 9–10; 10 representing the highest degree of satisfaction).

**Figure 5:**
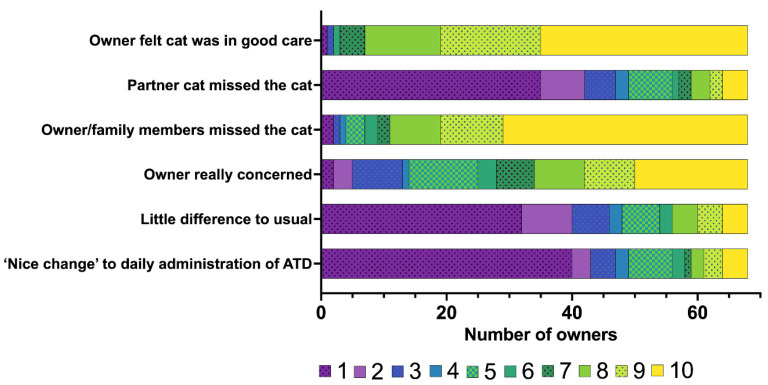
Bar chart summarising the owners’ experience at home during the hospitalisation of the 68 cats included in the study following radioiodine treatment, rated on a scale from 1 to 10 (1 = strongly disagree to 10 = strongly agree). The different numbers on the scale from 1 to 10 assigned by the owners are colour-coded (the legend for the colour scheme is within the figure). ATDs—antithyroid drugs.

Responses to questions concerning owners’ perceptions regarding their cat’s hospitalisation following RAIT are in Figure 5. During their cat’s hospitalisation, the owners of about half of the cats stated that they and their family missed the cat very much (owners of 39/68 [57%] cats rated 10/10; median: 10; IQR: 8–10), but they felt that their cat was in good care (owners of 33/68 [49%] cats rated 10/10; median: 9; IQR: 8–10) (Figure 5). Interestingly, only a few owners experienced the hospitalisation period as a ‘nice change’ to the daily administration of antithyroid medication—on the contrary, most owners strongly disagreed with this statement (owners of 40/68 cats rated 1/10, Figure 5).

Most cats could be reintegrated at home following hospitalisation for RAIT without any problem (*n* = 39/68 [57%] scored 1/10; median:1; IQR: 1–3), but the owner of one cat (*n* = 1/68; 1%) stated that the cat could not be reintegrated at all. The same owner noted that the other cat showed no signs of missing the treated cat during hospitalisation, which is not an uncommon answer in this study population (Figure 5, partner cat missed the cat: median: 1; IQR: 1–5).

**Figure 6:**
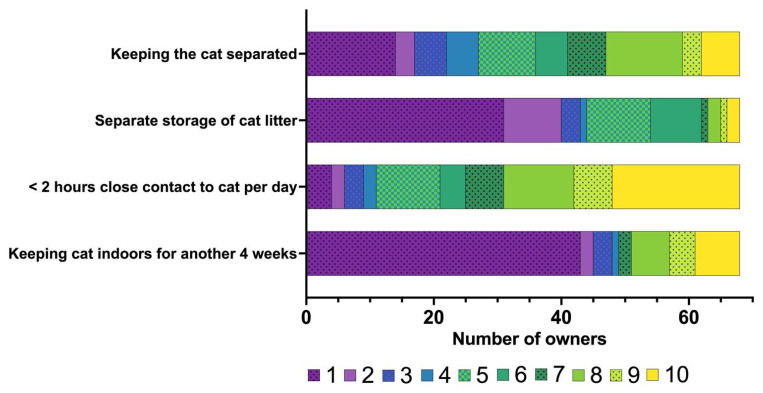
Bar chart summarising the owners’ perception of the burden imposed on them or their cats due to radiation safety measures following radioiodine treatment in the 68 cats included in the study. The owners rated their perception on a scale from 1 to 10 (1 = not a burden at all to 10 = extremely burdened). The different numbers on the scale from 1 to 10 assigned by the owners are colour-coded (the legend for the colour scheme is within the figure).

The owners experienced radiation safety measures that they had to keep for four weeks at home, following discharge from our hospital, as a similar level of burden for themselves (median: 5, IQR: 2–8) and their cats (median: 5, IQR: 2.8–8). The owners’ responses to questions concerning their perception of the specific radiation safety measures are summarised in Figure 6. The owners were most burdened by the requirement to limit the close contact with their cat to less than two hours per day (median: 8, IQR: 5–10), while keeping the cat indoors (median: 1; IQR: 1–7.3) or separate storage of the cat’s litter (median: 2, IQR: 1–5) were perceived as a minor problem (Figure 6).

After RAIT, most owners have experienced some improvement in their lives with their cat (median: 6; IQR: 2–8).

### 3.7. Re-Examinations Following RAIT and Support Given by Attending Clinicians During Follow-Up

**Figure 7:**
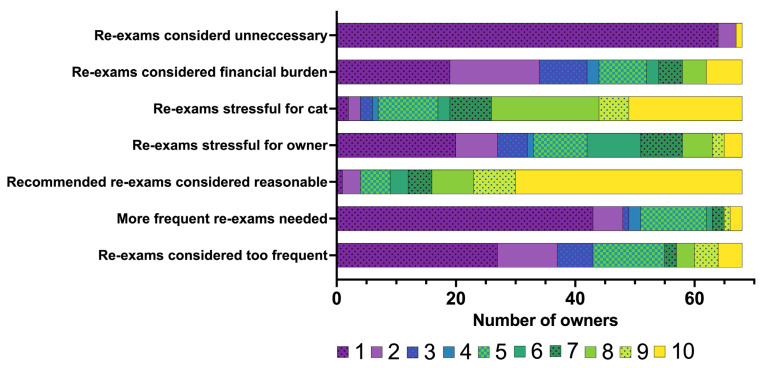
Bar chart summarising the owners’ perceptions of the recommended re-examinations after radioiodine treatment in the 68 cats included in the study. The owners rated their agreement with the statements concerning the re-examinations on a scale from 1 to 10 (1 = strongly disagree to 10 = strongly agree). The different numbers on the scale from 1 to 10 assigned by the owners are colour-coded (the legend for the colour scheme is within the figure).

Most owners were very satisfied with the care and advice provided by our clinic veterinarians during the follow-up period of six months post-RAIT (median: 9, IQR: 8–10; 10 highest degree of satisfaction). Nearly all owners stated that they performed the recommended re-examinations one month (*n* = 62/68; 91%) and three months (*n* = 62/68; 91%) after RAIT, while only two thirds (*n* = 46/68, 68%) had already presented their cat for the 6-month re-examination at the time of completing the questionnaire (10 additional cats [10/68] had their 6-month re-examination after completing the questionnaire). Almost one third (*n* = 19/68; 28%) of the owners presented their cat for additional re-examination(s) than those recommended by our clinic, and only one cat (*n* = 1/68; 1%) had no re-examinations at all after RAIT. The owners’ perception of the recommended re-examinations is summarised in Figure 7. Most owners perceived the recommended re-examinations as reasonable (median: 10, IQR: 8–10), neither too frequent (median: 2, IQR: 1–5), nor a financial burden (median: 2.5, IQR: 1–5) (Figure 7). Although the owners themselves did not perceive the re-examinations as particularly stressful (median: 5, IQR: 1–6.3), they felt that the re-examinations were stressful for their cats (median: 8, IQR: 5.75–10) (Figure 7).

### 3.8. Overall Satisfaction with RAIT

**Figure 8:**
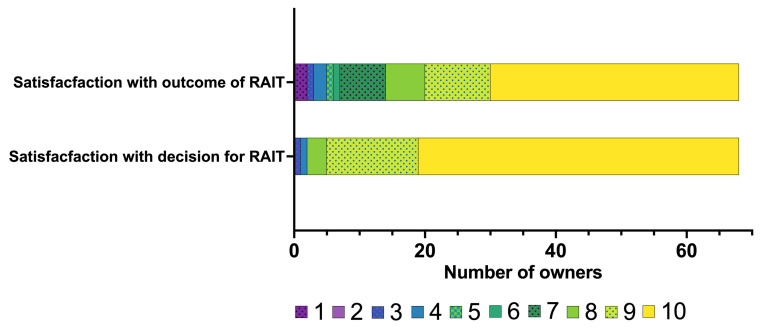
Bar chart summarising the owners’ satisfaction about their decision for and the outcome of radioiodine treatment (RAIT), rated on a scale from 1 to 10 (from 1 = not satisfied at all to 10 = extremely satisfied). The different numbers on the scale from 1 to 10 assigned by the owners are colour-coded (the legend for the colour scheme is within the figure).

Most owners were satisfied with their decision for their cat to undergo RAIT (median: 10, IQR: 9–10) as well as with the outcome of the RAIT (median: 10, IQR: 8–10) (Figure 8).

**Figure 9:**
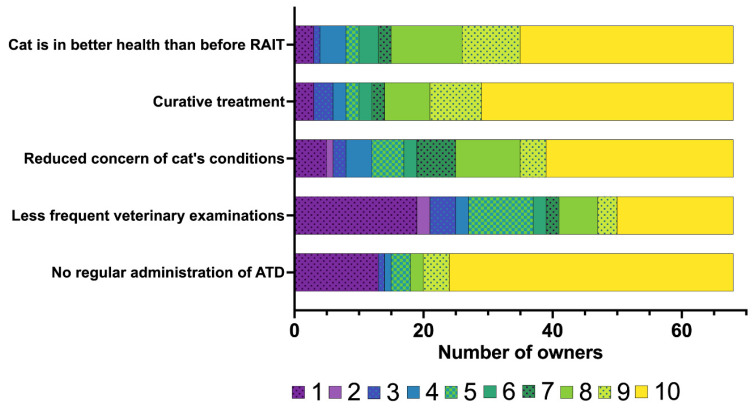
Bar chart summarising the main advantages of radioiodine treatment (RAIT) for owner and cat (*n* = 68), rated on a scale from 1 to 10 (1 = strongly disagree at all to 10 = strongly agree). The different numbers on the scale from 1 to 10 assigned by the owners are colour-coded (the legend for the colour scheme is within the figure).

**Figure 10:**
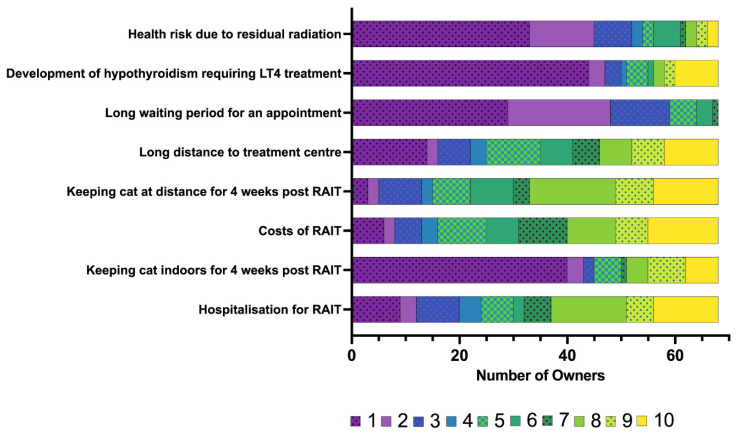
Bar charts summarising the main disadvantages of radioiodine treatment (RAIT) for owner and cat (*n* = 68), rated on a scale from 1 to 10 (1 = strongly disagree at all to 10 = strongly agree). The different numbers on the scale from 1 to 10 assigned by the owners are colour-coded (the legend for the colour scheme is within the figure). LT4—levothyroxine.

The owners’ responses to questions concerning their perception of the main advantages and disadvantages of RAIT are summarised in Figure 9 and Figure 10, respectively. Most owners agreed that the advantages of RAIT were that their cat was in better health than before (median: 9, IQR: 8–10), that the cat was cured of its hyperthyroidism (median: 10, IQR: 8–10), and that regular administration of ATDs was no longer necessary (median: 10, IQR: 5–10) (Figure 9). The opinion on the statement that their cat does not have to be checked so often by the veterinarian was mixed (median: 5, IQR: 1–10, Figure 9).

The owners perceived the need to keep their cat at a distance for another four weeks (median: 8, IQR: 5–9), the costs (median: 7, IQR: 5–9) and the hospitalisation after RAIT (median: 7, IQR: 3–8.3) as main disadvantages (Figure 10). Interestingly, the possible health risk due to residual radiation (median: 2, IQR: 1–3) and the development of hypothyroidism requiring levothyroxine treatment (median: 1, IQR: 1–4.3) were not perceived as disadvantages (Figure 10). However, the development of overt hypothyroidism was an important disadvantage for some owners (*n* = 8/68, 12% of owners rated 10/10; *n* = 2/68, 3% rated 9/10;).

Almost all owners (*n* = 65/68; 96%) would recommend RAIT to other owners of hyperthyroid cats. Three owners (*n* = 3/68; 4%) answered this question with unsure.

## 4. Discussion

To the authors’ knowledge, this is the first study investigating owners’ experiences and satisfaction with the treatment of hyperthyroid cats with RAIT in a prospective fashion. In addition to previous retrospective studies, we have also assessed changes in the owner’s perception of their cats’ HRQoL following RAIT using a validated QoL tool. Our study revealed that owners were satisfied with their decision for and the outcome of RAIT and that they were mostly concerned about the hospitalisation after RAIT because they missed their cat and were worried that their cat also missed them. During the first four weeks following RAIT, limiting close contact with their cat to a maximum of two hours per day was perceived as the radiation safety measure that imposed the most significant burden on the owners. The HRQoL significantly improved after RAIT, with no difference between cats that became euthyroid and those that became hypothyroid following RAIT.

The cohort of cats presented for RAIT at our hospital was similar to other studies, with European Shorthair being the most common breed (71%), most cats (64%) being senior (aged 11–14 years), and cats most commonly (42%) presented for RAIT within six months of diagnosis [8,10,16]. Similarly to previous studies [8,10], significant comorbidities were rare in our cohort, with almost half of the cats having no other disease than hyperthyroidism and dental disease being the most common concurrent condition (34%). This is most likely the result of a pre-selection by the referring veterinarians and clinicians at our hospital, as all referrals for RAIT are reviewed and confirmed by a group of clinicians with special interest in hyperthyroidism and RAIT. Such pre-selection is also common at other referral centres [18].

In agreement with previous retrospective studies examining owners’ perceptions of RAIT in the UK [8] and Belgium [10], most owners presented for RAIT at our hospital were informed about RAIT at our clinic by their primary veterinarian (64%) or found the information on the Internet (42%). However, a UK study on owner experiences and opinions regarding the management of hyperthyroid cats with ATDs found that 30% of owners were only offered medical treatment for their cats, with no other treatment options being discussed, and only 6% of owners had been offered an alternative treatment to ATDs [9]. This emphasises the importance of educating primary care veterinarians about the advantages and indications for RAIT and the expected outcomes. Furthermore, because the Internet is a common source of information for many owners, it is important to keep online resources/websites concerning RAIT up to date, providing scientifically sound and accurate information to enable owners to inform and educate themselves.

The most common reasons for owners of our study cats to choose RAIT were that it is the gold standard of hyperthyroidism treatment (35%), followed by side effects of the ATDs (23%), and cats not responding to ATDs (14%), which is in line with previous studies [8,10]. Because side effects of ATDs usually occur within the first few weeks or months of treatment [19], these cats are presented for RAIT fairly early in the course of their disease. On the other hand, cats not responding to ATDs might suffer from advanced-stage disease with severe hyperthyroidism caused by a large tumour (possibly carcinoma) [20] and might potentially have developed cardiovascular complications of the disease [21], making them high-risk patients in terms of sedation/anaesthesia (required for scintigraphy) as well as hospitalisation in isolation following RAIT. This emphasises the need for early owner (and veterinarian) education about RAIT as a treatment option for hyperthyroidism, which should not only be reserved for cases where ATDs failed. Owners should be informed by the veterinarians that hyperthyroidism is a progressive disease [20] and curative treatment options should be discussed from the outset of the treatment, unless contraindications exist (e.g., a severe comorbidity considerably reducing the cat’s life expectancy) [3].

Hospitalisation represented the strongest of all owners’ concerns regarding the RAIT, with a median score for this item of 9 out of 10 (score 10 representing the highest level of concern) and owners of 35% of cats giving a score of 10. Hospitalisation was also perceived as one of the main disadvantages of RAIT. In the authors’ clinic, the cats are hospitalised for five to ten days following RAIT (rarely, cats receiving a high dose of iodine of up to 30 mCi for the treatment of a suspected thyroid carcinoma [15] might need to be hospitalised for longer time periods). Interestingly, in Belgium, the owners also rated hospitalisation as the most stressful factor regarding the RAIT even though the duration of hospitalisation in Belgium was shorter (five days vs. five to ten days in our clinic) [10]. Therefore, it might not necessarily be the length of hospitalisation but rather the requirement for hospitalisation itself that is the cause for concern. In human medicine, it has been described that those people who own a pet might be reluctant to separate from their pet, leading to non-compliance of pet owners concerning their own health, e.g., avoiding medical care out of fear of being admitted to the hospital and therefore separated from their pet [22]. Accordingly, the concern about the hospitalisation of their cat could potentially lead to a decision against RAIT, out of fear of separation, although this could not be assessed in our study as only owners who chose RAIT were surveyed.

The owners of our study cats were worried that their cat might feel lonely during the hospitalisation and might miss them and that they would miss their cat (all three items rated equally with a median score of 9 out of 10, with score 10 representing the strongest concern). These concerns reflect the strong emotional bond between owners and their cats and highlight the similarities between the owner-cat relationship and the parent–child relationship [23,24]. Therefore, owner concerns must be taken seriously and should be addressed during owner counselling prior to RAIT and during the hospitalisation. For example, providing detailed information about the hospitalisation facilities, including photographs of the kennels, might reassure owners that their cat will have enough opportunities to rest and hide if he/she prefers. Hiding was found to be an important coping mechanism for cats dealing with an uncontrollable and unpredictable environment [25]. Implementation of environmental enrichment strategies might also help to reduce stress levels in cats [26]; therefore, besides providing scratching posts in the kennels, in our hospital, the owners are also allowed to bring their cat’s own toy(s). Furthermore, daily phone call updates by the attending clinician are another way of reassuring owners that their cat is well, which was reflected by owners’ high ratings of their satisfaction with information provided during hospitalisation (median score 10 out of 10, with 10 representing the highest level of satisfaction). It might also be comforting for the owners that cats hospitalised for elective procedures were found to adapt rapidly to their new environment [27]. This was shown in a small prospective study assessing demeanour score, appetite and litter tray usage during hospitalisation for neutering that found that once the cats realised that their new environment was harmless, they could express normal feeding and urination behaviour. Although the study was conducted with young cats (1–2 years old) [27], and the information might therefore not be completely transferrable to older hyperthyroid cats, it is the subjective impression of the authors that most cats adapt well to the hospital environment, especially in the isolation ward, where the kennels are larger, allowing for hiding, and the area is quieter, separated from the remaining busy hospital rooms. Addressing owners’ worries and concerns, as discussed above, as well as making them understand more clearly why the hospitalisation and separation are important radiation safety measures needed to protect themselves and their families, could alleviate owners’ anxiety concerning the hospitalisation for the RAIT.

Interestingly, prior to RAIT, owners were less concerned about the length of travel to our hospital (median score of 4 out of 10, with 1 being no concern at all), even though most had to travel more than 100 km, and a few lived more than 400 km away. Similarly, in the UK study, the distance to the referral clinic also had only a minor impact on the decision to pursue RAIT [8]. It should, however, be noted that in our study only owners who opted for RAIT were surveyed, and it therefore could not be evaluated what proportion of owners might decide against RAIT because of the lengthy travel. The costs of the treatment (median score of 4.5 out of 10, with 1 being no concern at all) also did not seem to represent a major worry in our or the previous studies [8,10], despite the fact that in our study only three cats (4%) had health insurance that covered the costs of the treatment, which is similar to a study carried out in Belgium, where 3.4% of the cats were insured [10]. On the other hand, it should be mentioned that while the upfront costs of RAIT are high, the cumulative costs of treatment with ATDs or feeding an LID over the cat’s lifetime are approximately equivalent, and therefore, in the long term, the overall costs are comparable. However, with ATDs or an LID, the expenses are stretched over time [4].

Although one might expect the opposite, the owners barely worried about the effects of possible residual radiation from their cat on their own health, with owners of 50% of the cats having no concerns at all (median score of 1.5 out of 10, with 1 being no concern at all). Personal health risk due to residual radiation was also not considered any important disadvantage of RAIT by the owners of our study cats. These findings are similar to the previously mentioned UK study, where the concern about potential human health risks due to radiation also only had a low impact on the treatment decision [8]. This low level of concern about residual radiation might be partly because owners who worry about radiation do not choose RAIT in the first place. However, another reason might be that owners underestimate the risk because it is an invisible hazard with no immediately visible consequences. Furthermore, following RAIT, the radiation safety measures generally were not perceived as a particularly strong burden for owners (median score 5 out of 10, with 10 representing the strongest burden), with having to limit the close contact to their cat to two hours per day considered the only significant burden. This, again, is likely reflecting the perceived disruption of the owner-animal bond [23,24] as discussed in relation to the hospitalisation for RAIT above. However, because we cannot be absolutely certain to what extent the owners abide by the radiation protection measures, the rather low level of burden by some measures, such as the separate storage of the used cat litter, might also be due to low compliance with this particular requirement. Keeping the cats post-RAIT indoors might also have been unproblematic in our study cohort because most cats were either strictly kept indoors or only allowed outdoors for limited periods of time. The owners of cats that cannot be kept indoors for four weeks are unlikely to opt for RAIT in the first place.

The frequency of re-examinations following RAIT that are suggested by our clinic is in line with recommendations given by others [17]. For the purpose of consistency of laboratory assessments, we routinely offer the owners to have the blood tests performed via our clinic and receive advice (e.g., regarding the need for LT4 supplementation or when to start a kidney diet) from our team of clinicians specialised in hyperthyroidism and RAIT. As shown by the responses to our survey, most owners considered the number of re-examinations and the recommended intervals reasonable and were very satisfied with the advice provided by our clinicians (median score 9 out of 10, with 10 representing the highest level of satisfaction). Although a number of owners perceived the re-examinations as a source of stress for their cats, the compliance still was high, with approximately 90% of owners performing the first two re-examinations and nearly 70% presenting their cat for the 6-month re-examination. Barely any owners considered the re-examinations unnecessary, and only a single owner did not perform any re-examinations at all. A possible explanation for this high level of compliance is that RAIT is likely chosen by owners who are particularly concerned about and well-informed regarding their cat’s health. Furthermore, the high level of satisfaction with the information and support provided by our clinicians before the RAIT, during the hospitalisation and after RAIT indicates that although initial concerns are common among owners, satisfaction with the provided care might be the primary factor in maintaining compliance [28] and alleviating the worry about their pets. Previous research has shown that owner compliance can be improved through a good relationship between owner and veterinarian [29]. As most owners of our study cats also participated in other research projects run by our clinic, related to the re-examination and follow-up after RAIT, they knew the team of our clinicians and their primary contact person well, which might have also improved their compliance.

Our study revealed that the owners were extremely satisfied with their decision for their cat to undergo RAIT and with its outcome, with the median score for both items being 10 out of 10 (with 10 representing extreme satisfaction). The owners generally agreed that the improved health of their cat, the cure of the hyperthyroidism and the resulting reduced level of concern for their cat’s condition were the main advantages of RAIT. Although the reduction in the frequency of veterinary re-examinations and the cessation of regular tablet administration were also considered as advantages of RAIT by some, a number of owners disagreed. Although the frequency of re-examinations might be lower than that required for poorly controlled hyperthyroid cats, regular check-ups are still recommended, especially within the first six months of RAIT [16,17]. Therefore, this advantage might only become apparent at a later stage and was not captured in our study due to the follow-up being limited to six months post treatment. Furthermore, as some cats in this study developed hypothyroidism and required medication, the argument that they required fewer tablets after RAIT was not a valid advantage for these owners.

A number of disadvantages were presented to the owners for their consideration and evaluation. The health risk because of residual radiation (discussed above), the distance to the referral clinic (discussed above), the waiting period for an appointment and the development of hypothyroidism were only perceived as minor disadvantages. Most owners only had to wait a few weeks to a couple of months for an appointment, which might not be considered long given the highly specialised treatment, which is only offered at one additional facility in Germany. Although 21/68 cats developed hypothyroidism requiring LT4 replacement, and therefore ongoing administration of medication was needed following RAIT, this was not perceived as a major disadvantage by most owners. This might be because our study cats did not develop any significant clinical signs of hypothyroidism (likely due to early start of LT4 supplementation), which usually are subtle in comparison to the pronounced clinical signs of hyperthyroidism [30]. Also, HRQoL improved in all cats with resolution of hyperthyroidism following RAIT, irrespective of achieving eu- or hypothyroidism.

Because owners’ perceptions about treatment outcomes and their satisfaction might be affected by their perception of their cats’ clinical signs and QoL, these were also analysed in our study. Clinical signs improved significantly within the first six months after RAIT, with 76% of owners reporting noticeable improvement within the first two months after RAIT. These findings are in agreement with previous studies, demonstrating the rapid clinical improvement of hyperthyroid cats after RAIT [2]. This finding deserves attention, given that 44% of our study cats already were euthyroid before RAIT, indicating that RAIT provides additional therapeutic benefits even in cases where medical management had already achieved disease control. An alternative explanation for this result could be the placebo effect, i.e., that the owners want their cat to feel better and that this has an influence on the owner’s perception of the cat’s symptoms.

Alongside improvement in clinical signs, HRQoL also significantly improved between enrolment and six months after RAIT. These findings correspond with our previous study that found better QoL in RAI-treated cats when compared to cats receiving ATDs [3,31]. Interestingly, although 38% of the cats with resolution of hyperthyroidism following RAIT became hypothyroid, no significant difference in HRQoL was observed between euthyroid and hypothyroid cats. This indicates that, despite the occurrence of iatrogenic hypothyroidism, the overall QoL post-treatment was still significantly better than before RAIT. As discussed above, the lack of clinical signs of overt hypothyroidism, likely due to early institution of LT4 replacement, might explain why HRQoL of hypothyroid cats was no different from euthyroid. The same has already been shown in older people, where subclinical and overt hypothyroidism have been shown not to reduce HRQoL [32,33]. Therefore, early detection and treatment of iatrogenic hypothyroidism might help prevent any negative impact on HRQoL. This highlights the critical importance of thorough owner education prior to RAIT. Owners must be informed that their cat might develop iatrogenic hypothyroidism following treatment (or, rarely, remain hyperthyroid), necessitating medication [2]. Such education is essential for managing expectations and ensuring owners understand the potential need for lifelong medication (or re-treatment).

Our study has some limitations. First, since the owners were aware of the purpose of the study, they might have been inclined to choose answers that align with their perceived opinion of their cats’ QoL, which could have impacted the responses [34]. As with any non-blinded questionnaire study, observer bias is a potential issue [35]. Owners might alter their perception or reporting of their cat’s clinical signs due to their involvement in the study, knowing their responses will be analysed. They might also have been reluctant to provide negative feedback [35]. The comorbidities were only owner-reported and not confirmed by the medical records. Therefore, they might not reflect the true condition of the enrolled cats. Despite using a validated QoL tool in this study, the perceived improvement in QoL after RAIT could have been influenced by the owners’ wish for a positive outcome, leading them to report answers in a biassed manner, possibly overstating improvements. Furthermore, the analysis was limited to owners who were willing to participate, which might have led to respondents with strong opinions or a deeper interest in this topic. Also, the second questionnaire (six months after RAIT) might have been too delayed to capture the experiences of the owners during the hospitalisation, and questions regarding hospitalisation, reintegration and radiation safety measures could have been affected by a recall bias [36]. Nevertheless, we chose this timepoint to allow sufficient time for the normalisation/stabilisation of thyroid function and therefore improvement of QoL after RAIT but opted not to send another follow-up questionnaire at an earlier timepoint (e.g., to better capture experiences with hospitalisation) to avoid overstretching owner compliance. Another limitation is that all questions were closed-ended, restricting response variety and preventing owners from fully expressing their opinions or suggesting changes [37]. Additionally, the predefined options can influence responses and restrict the discovery of unexpected insights [37]. While this method limits the insights, it was chosen to reduce missing data and simplify analysis. Additionally, not all questionnaires were answered at exactly the same timepoint following treatment. In particular, some owners had already completed the 6-month re-examination and were aware of their cat’s thyroid status and bloodwork, while others completed the questionnaire before the re-examination.

## 5. Conclusions

This study provides insights into owner experiences and satisfaction with RAIT, revealing high levels of satisfaction with both the decision for RAIT and its outcome. Hospitalisation was the main owner concern regarding RAIT, and having to limit close contact with their cat to a maximum of two hours per day was perceived as the radiation safety measure that imposed the most significant burden on the owners. Importantly, HRQoL significantly improved after RAIT, with no difference between eu- and hypothyroid cats six months after RAIT. Besides the identification of positive effects of RAIT on the HRQoL, this study highlights the importance of owner education and clear veterinary guidance to facilitate informed decision-making and optimise long-term treatment outcomes.

## Figures and Tables

**Table 1:** Age, sex and breed of 78 hyperthyroid cats presented for radioiodine treatment and included in the study.

Parameter	Choice of Options	Number *n* (%)
Age	3 to 6 years	2 (3)
	7 to 10 years	14 (18)
	11 to 14 years	50 (64)
	Over 15 years	11 (14)
	Unknown	1 (1)
Sex	Male	0 (0)
	Male, neutered	41 (53)
	Female	1 (1)
	Female, neutered	36 (46)
Breed	European Shorthair	55 (71)
	Mixed Breed	7 (9)
	Norwegian Forest	4 (5)
	Maine Coon	2 (3)
	British Shorthair	2 (3)
	Persian	1 (1)
	Ragdoll	1 (1)
	Others	6 (8)

**Table 2:** Information about the duration and management of the cats’ hyperthyroidism and the presence of comorbidities prior to the presentation for radioiodine treatment (RAIT) in 78 cats included in the study.

Parameter	Choice of Options	Number *n* (%)
Duration of hyperthyroidism	6 months or less	33 (42)
	7 to 12 months	19 (25)
	1 to 2 years	16 (21)
	3 years or longer	6 (8)
	Unknown	4 (5)
Treatment before RAIT	Antithyroid drugs	57 (73)
	Iodine-reduced diet	4 (5)
	RAIT	2 (3)
	No treatment	15 (19)
Thyroid status prior to RAIT	Hyperthyroid	34 (44)
	Euthyroid	34 (44)
	Hypothyroid	2 (3)
	Unknown	8 (10)
Frequency of thyroid hormone evaluation	Four times a year	23 (29)
	Three times a year	15 (19)
	Twice a year	4 (5)
	Once a year	8 (10)
	Other	28 (36)
Comorbidities	No other disease	37 (47)
	Dental disease	27 (34)
	Musculoskeletal disease	9 (11)
	Heart disease	7 (8)
	Respiratory tract disease	5 (6)
	Chronic kidney disease	2 (3)
	Gastrointestinal disease	2 (3)
	Skin disease	2 (3)
	Urinary tract disease	1 (1)
	Other	15 (18)
Treatment for comorbidities	No treatment	57 (73)
	Oral medication	14 (17)
	Special diet	3 (4)
	Medical treatment with regular injections	2 (3)
	Other	2 (3)

**Table 3:** Overview of the main reasons why the owners chose radioiodine treatment (RAIT), how they learned about the possibility of RAIT at our clinic, the travel distance and the waiting period for the appointment in the 78 study cats. Please note that the question concerning how the owners obtained information about the RAIT allowed for multiple answers; therefore, the answers do not add up to 100%.

Parameter	Choice of Options	Number *n* (%)
Main reason for choosing RAIT	Because RAIT is the treatment of choice (“gold standard”).	27 (35)
	The cat has side effects from the antithyroid drugs.	18 (23)
	The owner wanted RAIT since the cat was diagnosed.	11 (14)
	The cat does not respond to antithyroid drugs.	7 (9)
	Advice from primary veterinarian.	5 (6)
	Difficulties administering antithyroid medication.	3 (4)
	Other reasons.	7 (9)
How the owners learned about the possibility of RAIT at the referral clinic	Advice from primary care veterinarian.	50 (64)
	Internet.	33 (42)
	Social media.	8 (10)
	Owner already owned a cat treated with RAIT.	1 (1)
Travel distance to the referral clinic	Less than 100 km.	16 (21)
	100 to 200 km.	27 (35)
	200 to 300 km.	18 (23)
	300 to 400 km.	8 (10)
	More than 400 km.	9 (12)
Waiting period for the appointment	Less than 1 month.	23 (29)
	1 to 2 months.	44 (56)
	3 to 4 months.	26 (33)
	5 to 6 months.	6 (8)

**Table 4:** Comparison of the cats’ clinical signs (*n* = 68) (rated on a 10-point scale; 1 = not present, 10 = very strong), the health-related quality of life (HRQoL) and owners’ general concern of their cats’ hyperthyroidism (rated on a 10-point scale; 1 = not a concern for me at all, 10 = concerns me strongly) upon enrolment (prior to radioiodine treatment [RAIT]) and six months following RAIT.

	Prior to RAIT		Six Months After RAIT		*p*-Values
	Median	IQR	Median	IQR	
Weight loss	5	3–8	1	1–1	<0.01
Muscle wasting	3	1–6	1	1–2	<0.01
Restlessness	5	2–2.8	2	1–3	<0.01
Aggression	1	1–3.8	1	1–2	<0.01
Vomiting	3	1–5.8	1	1–3	<0.01
Diarrhoea	2	1–3	1	1–2	0.04
Polyuria	4	1–6	1.5	1–3	<0.01
Polydipsia	4	2–7	2	1–3.3	<0.01
Poor coat quality	3	2–7	1	1–2	<0.01
HRQoL-Score	92.3	67.3–123.5	66.5	50.8–89.9	<0.01
General concern about the cat’s hyperthyroidism	9	7–10	2	1–4	<0.01

**Table 5:** Information about new comorbidities and the required treatment 6 months after radioiodine treatment in 68 cats included in the study. Please note that the question concerning the new comorbidities and their treatment allowed for multiple answers; therefore, the answers do not add up to 100%.

Parameter	Choice of Options	Number *n* (%)
Comorbidities	No other disease	35 (51)
	Chronic kidney disease	10 (15)
	Heart disease	2 (3)
	Musculoskeletal disease	1 (1)
	Skin disease	1 (1)
	Other	10 (15)
Treatment for comorbidities	No treatment	40 (59)
	Oral medication	12 (18)
	Special diet	5 (7)
	Medical treatment with regular injections	1 (1)
	Other	10 (15)

## Data Availability

The data presented in this study are available on request from the corresponding author.

## Supplementary Materials

The following supporting information can be downloaded at: https://www.mdpi.com/article/10.3390/vetsci12050458/s1, S1: First questionnaire (to be completed within the week before RAIT); S2: Second questionnaire (to be completed six months after RAIT); S3: Socio-demographic data about the owners (*n* = 77).

## Abbreviations

The following abbreviations are used in this manuscript:

ATDs	Antithyroid drugs
HRQoL	Health-related quality of life
IQR	Interquartile range
LID	Low iodine diet
LT4	Levothyroxine
QoL	Quality of life
RAI	Radioiodine
RAIT	Radioiodine treatment
RI	Reference interval
TSH	Thyroid stimulating hormone
TT4	Total thyroxine
