# Supplementary material for: Owners’ Experience and Satisfaction with Radioiodine Treatment in Hyperthyroid Cats—A Prospective Questionnaire Study
Source: Vet Sci. 2025 May 10;12(5):458. doi: 10.3390/vetsci12050458 (PMC12116025; doi:10.3390/vetsci12050458)
Supplement: Supplementary file 1 [file vetsci-12-00458-s001.zip › Supplementary.docx]

1. First questionnaire (to be completed within the week before RAIT)

| Question | Answer sets |
| --- | --- |
| CAT RELATED QUESTIONS | |
| 1. What is the name of your cat? | _______________________________________________ |
| 2. How old is your cat? | - Less than 6 months - 7 months to 2 years - 3 to 6 years - 7 to 10 years - 11 to 14 years - Over 15 years - Unknown |
| 3. What is the sex of your cat? | - Female - Female neutered - Male - Male neutered |
| 4. What breed is your cat? | - Domestic Shorthair/ European Shorthair - Maine Coon - Norwegian Forest - British Shorthair - Bengal - Siamese - Persian - Ragdoll - Mixed breed - Unknown - Other: _______________________ |
| 5. How long have you had your cat? | - Less than 6 months - 7 to 12 months - 1 to 2 years - 2 to 4 years - 5 to 10 years - For more than 10 years |
| 6. Is your cat an outdoor cat? | - No, only on the leash. - Only in the garden and under supervision. - Yes, my cat is an outdoor cat, but does come into the house. - Yes, my cat is an outdoor cat and never comes into the house. |
| 7. For how long has your cat been hyperthyroid?  *Please state the time in months or years.* | _____________________________________________ |
| 8. How pronounced/ severe are the following symptoms in your cat?  *Please rate the symptoms on a scale from 1= not present to 10= very strong.*   \|  \| 1 \| 2 \| 3 \| 4 \| 5 \| 6 \| 7 \| 8 \| 9 \| 10 \| \| --- \| --- \| --- \| --- \| --- \| --- \| --- \| --- \| --- \| --- \| --- \| \| Weight loss \| o \| o \| o \| o \| o \| o \| o \| o \| o \| o \| \| Muscle wasting \| o \| o \| o \| o \| o \| o \| o \| o \| o \| o \| \| Restlessness \| o \| o \| o \| o \| o \| o \| o \| o \| o \| o \| \| Aggression \| o \| o \| o \| o \| o \| o \| o \| o \| o \| o \| \| Vomiting \| o \| o \| o \| o \| o \| o \| o \| o \| o \| o \| \| Diarrhoea \| o \| o \| o \| o \| o \| o \| o \| o \| o \| o \| \| Increased urination \| o \| o \| o \| o \| o \| o \| o \| o \| o \| o \| \| Increased thirst \| o \| o \| o \| o \| o \| o \| o \| o \| o \| o \| \| Poor coat quality \| o \| o \| o \| o \| o \| o \| o \| o \| o \| o \| | |
| 9. How concerned are you about your cat’s hyperthyroidism?  *Please rate the following issues on a scale from 1 = not a concern for me at all to 10= concerns me strongly.*   \| 1 \| 2 \| 3 \| 4 \| 5 \| 6 \| 7 \| 8 \| 9 \| 10 \| \| --- \| --- \| --- \| --- \| --- \| --- \| --- \| --- \| --- \| --- \| \| o \| o \| o \| o \| o \| o \| o \| o \| o \| o \| | |
| 10. What treatment options for hyperthyroidism are you aware of?  *Please select all options you have already heard of before you started this survey.* | - Antithyroid tablets or syrup (e.g., Felimazole, Thiamatab, Vidalta, Thyronorm, …) - Antithyroid ear ointment - Iodine reduced food (e.g.: Hills y/d) - Radioiodine treatment - Surgery/thyroidectomy - No treatment - Other: ______________________________ |
| 11. What treatment is your cat currently receiving for his/ her hyperthyroidism? | - Antithyroid tablets or syrup (e.g., Felimazole, Thiamatab, Vidalta, Thyronorm, …) - Antithyroid ear ointment - Iodine reduced food (e.g.: Hills y/d) - Radioiodine treatment - Surgery/ thyroidectomy - No treatment - Other: _______________________________ |
| 12. What was your main reason for choosing this treatment option? | - Advice from primary care veterinarian - Internet research - Personal advice (non-veterinarian) from a forum / discussion group - Advice from a friend - Own experience (my other cat has/had hyperthyroidism) - Own experience (I suffer from hyperthyroidism myself) - No treatment - Other: ____________________________________ |
| 13. For how long has your cat been receiving treatment for his/her hyperthyroidism? (In case of radioiodine treatment or thyroidectomy, please state how long ago this treatment was carried out.) | - Less than 1 months - 2 to 3 months - 4 to 6 months - 7 to 12 months - For about 1 year - For about 2 years - For about 3 years - For about 4 years - For about 5 years or longer - No treatment - Other: ____________________________________ |
| 14. Does your cat have normal thyroid levels at the moment? | - Yes - No, the thyroid hormone levels are increased. - No, the thyroid hormone levels are decreased. - I don't know. |
| 15. Has your cat been diagnosed with a condition other than hyperthyroidism at the moment? | - No other disease - Chronic nephropathy (kidney disease) - Hypertension - Diabetes mellitus - Disease of the musculoskeletal system (arthritis, chronical lameness etc.) - Disease of the skin (including ear disease, e.g., otitis) - Disease of the gastro-intestinal tract (chronic enteropathy, chronic pancreatitis etc.) - Dental disease - Respiratory tract disease (feline rhinitis, feline bronchial disease/ asthma) - Disease of the urinary tract (bladder stones, chronic cystitis) - Feline immunodeficiency (FIV) - Feline leukaemia virus (FeLV) - Heart Disease - Other: _____________________ |
| 16. If your cat suffers from any of the above-mentioned diseases, what treatment is currently required? | - Oral medication (e.g., tablets) - Medical treatment with regular injections - Special diet - No current treatment - Other: _____________________ |
| 17. How often are the thyroid hormone levels of your cat being checked at the vet? | - Four times a year (every 3 months) - Three times a year (every 4 months) - Twice a year (every 6 months) - Once a year - Other: _____________________ |
| OWNER RELATED QUESTIONS | |
| 1. What year were you born? | _____________________ |
| 2. Please specify your gender. | - Male - Female - Divers - Prefer not to say - Other |
| 3. In which country are you currently resident? | _____________________ |
| 4. Are there any children living together with the cat? If yes, how old is the youngest child? | - No - Younger than 3 years - 3 to 6 years - 7 to 12 years - 13 to 18 years - Older than 18 years - Prefer not to say - Other: _____________________ |
| 5. What is your highest level of education? | - Secondary education - Bachelor’s degree - Master’s degree - State examination - Doctorate (PhD) - Prefer not to say - Other: |
| 6. What is your current work situation? | - Employed full-time - Employed part-time - Employed full-time (work from home) - Employed full-time (hybrid) - Unemployed (seeking work) - Unemployed (not seeking work) - Retired - Unable to work - Pupil - Student - Apprentice / trainee - Prefer not to say - Other: _____________________ |
| 7. Who cares mainly for your cat? | - You - Your partner - Other family members - Neighbors - Prefer not to say - Other: _____________________ |
| 8. How much time do you and your cat spend together in direct proximity (less than 2 meters apart)? | - I spend ____ hours per day with my cat. (Please estimate the time) - I don’t see my cat every day. - Other: _____________________ |
| 9. Is this cat your first cat? | - Yes - No |
| 10. Have you ever owned a cat with hyperthyroidism before? | - Yes - No |
| 11. How many cats do you own? | _____________________ |
| QUESTIONS PRIOR TO RADIOIODINE TREATMENT | |
| 1. Why did you choose radioiodine treatment for your cat? | - My cat has severe side effects of the antithyroid drugs (tablets, syrup or ear ointment) - I have difficulties administering the antithyroid medication. - My cat doesn’t respond to the antithyroid medication. - Because radioiodine treatment is the treatment of choice (‘gold standard’). - I wanted radioiodine treatment for my cat since he/she was diagnosed with hyperthyroidism. - Other: |
| 2. How did you learn about the option for radioiodine treatment at the veterinary clinic of the Justus-Liebig-University in Giessen (Germany)? | - Advice from primary care veterinarian - Advice from a friend whose cat was treated with radioiodine treatment - My other cat was treated with radioiodine treatment - Pet journal - Social media - Internet - Other |
| 3. How far is it for you to the veterinary clinic of the Justus-Liebig-University in Giessen (Germany)? | - Less than 100 km - 100 to 200 km - 200 to 300 km - 300 to 400 km - Over 400 km |
| 4. How long did you wait for your appointment? | - Less than 1 month - 1 to 2 months - 3 to 4 months - 5 to 6 months - Longer than 7 months - Other: |
| 5. Does your cat have insurance and does it cover the cost of the radioiodine treatment? | - Yes - No |
| 6. Which of the following issues is concerning you regarding the radioiodine treatment? *Please rate the following issues on a scale from 1= not a concern for me at all to 10= concerns me strongly.*   \|  \| 1 \| 2 \| 3 \| 4 \| 5 \| 6 \| 7 \| 8 \| 9 \| 10 \| \| --- \| --- \| --- \| --- \| --- \| --- \| --- \| --- \| --- \| --- \| --- \| \| The long distance to the treatment facility. \| o \| o \| o \| o \| o \| o \| o \| o \| o \| o \| \| The cost of the radioiodine treatment. \| o \| o \| o \| o \| o \| o \| o \| o \| o \| o \| \| The anaesthetic risk. \| o \| o \| o \| o \| o \| o \| o \| o \| o \| o \| \| The hospitalization after the radioiodine treatment. \| o \| o \| o \| o \| o \| o \| o \| o \| o \| o \| \| Having to keep your cat indoor for another 4 weeks after radioiodine treatment. \| o \| o \| o \| o \| o \| o \| o \| o \| o \| o \| \| The possible health risk for you and your family due to the residual radiation (e.g. when handling the litterbox) \| o \| o \| o \| o \| o \| o \| o \| o \| o \| o \| | |
| 7. Which of the following issues is concerning you regarding the hospitalization of your cat for radioiodine treatment? *Please rate the following issues on a scale from 1= not a concern for me at all to 10= concerns me strongly.*   \|  \| 1 \| 2 \| 3 \| 4 \| 5 \| 6 \| 7 \| 8 \| 9 \| 10 \| \| --- \| --- \| --- \| --- \| --- \| --- \| --- \| --- \| --- \| --- \| --- \| \| That your cat might not eat. \| o \| o \| o \| o \| o \| o \| o \| o \| o \| o \| \| That your cat might miss you. \| o \| o \| o \| o \| o \| o \| o \| o \| o \| o \| \| That you will miss your cat. \| o \| o \| o \| o \| o \| o \| o \| o \| o \| o \| \| That you can’t visit your cat. \| o \| o \| o \| o \| o \| o \| o \| o \| o \| o \| \| That your cat can’t go outdoors. \| o \| o \| o \| o \| o \| o \| o \| o \| o \| o \| \| That only limited monitoring is allowed following radioiodine treatment. \| o \| o \| o \| o \| o \| o \| o \| o \| o \| o \| | |
| 8. How satisfied were you with the information provided by the radioiodine team of our clinic before radioiodine treatment?  *Please rate on a scale from 1= not satisfied at all to 10= very satisfied.*   \| 1 \| 2 \| 3 \| 4 \| 5 \| 6 \| 7 \| 8 \| 9 \| 10 \| \| --- \| --- \| --- \| --- \| --- \| --- \| --- \| --- \| --- \| --- \| \| o \| o \| o \| o \| o \| o \| o \| o \| o \| o \| | |

1. **Second questionnaire (to be completed six months after RAIT)**

| Question | | Answer sets | |
| --- | --- | --- | --- |
| CAT RELATED QUESTIONS | | | |
| 1. What is the name of your cat? | | _______________________________________________ | |
| 2. How pronounced/ severe are the following symptoms in your cat at the moment?  *Please rate the symptoms on a scale from 1= not present to 10= very strong.*   \|  \| 1 \| 2 \| 3 \| 4 \| 5 \| 6 \| 7 \| 8 \| 9 \| 10 \| \| --- \| --- \| --- \| --- \| --- \| --- \| --- \| --- \| --- \| --- \| --- \| \| Weight loss \| o \| o \| o \| o \| o \| o \| o \| o \| o \| o \| \| Muscle wasting \| o \| o \| o \| o \| o \| o \| o \| o \| o \| o \| \| Restlessness \| o \| o \| o \| o \| o \| o \| o \| o \| o \| o \| \| Aggression \| o \| o \| o \| o \| o \| o \| o \| o \| o \| o \| \| Vomiting \| o \| o \| o \| o \| o \| o \| o \| o \| o \| o \| \| Diarrhoea \| o \| o \| o \| o \| o \| o \| o \| o \| o \| o \| \| Increased urination \| o \| o \| o \| o \| o \| o \| o \| o \| o \| o \| \| Increased thirst \| o \| o \| o \| o \| o \| o \| o \| o \| o \| o \| \| Poor coat quality \| o \| o \| o \| o \| o \| o \| o \| o \| o \| o \| | | | |
| 3. Does your cat have normal thyroid levels at the moment? | | - Yes - No, the thyroid hormone levels are increased. - No, the thyroid hormone levels are decreased. - I don't know. | |
| 4. Has your cat been diagnosed with a condition other than hyperthyroidism after radioiodine treatment? | | - No other disease - Hypothyroidism - Chronic nephropathy (kidney disease) - Hypertension - Diabetes mellitus - Disease of the musculoskeletal system (arthritis, chronical lameness etc.) - Disease of the skin (including ear disease, e.g., otitis) - Disease of the gastro-intestinal tract (chronic enteropathy, chronic pancreatitis etc.) - Dental disease - Respiratory tract disease (feline rhinitis, feline bronchial disease/ asthma) - Disease of the urinary tract (bladder stones, chronic cystitis) - Feline immunodeficiency (FIV) - Feline leukaemia virus (FeLV) - Heart disease - Other | |
| 5. If your cat suffers from any of the above-mentioned diseases, what treatment is currently required? | | - Oral medication (e.g., tablets) - Medical treatment with regular injections - Special diet - No current treatment - Other: _____________________ | |
| 6. How concerned are you about your cat’s hyperthyroidism at the moment?  *Please rate the following issues on a scale from 1 = not a concern for me at all to 10= concerns me strongly.*   \| 1 \| 2 \| 3 \| 4 \| 5 \| 6 \| 7 \| 8 \| 9 \| 10 \| \| --- \| --- \| --- \| --- \| --- \| --- \| --- \| --- \| --- \| --- \| \| o \| o \| o \| o \| o \| o \| o \| o \| o \| o \| | | | |
| QUESTIONS 6 MONTHS AFTER RADIOIODINE TREATMENT | | | |
| 1.Satisfaction  *Please rate the following issues on a scale from 1 = not satisfied at all to 10 = very satisfied.*   \|  \| 1 \| 2 \| 3 \| 4 \| 5 \| 6 \| 7 \| 8 \| 9 \| 10 \| \| --- \| --- \| --- \| --- \| --- \| --- \| --- \| --- \| --- \| --- \| --- \| \| How satisfied were you with the information provided by our veterinarians prior to the radioiodine treatment? \| o \| o \| o \| o \| o \| o \| o \| o \| o \| o \| \| How satisfied were you with the care and support of our veterinarians during the hospitalization of your cat? \| o \| o \| o \| o \| o \| o \| o \| o \| o \| o \| | | | |
| 2. How was your experience at home while your cat was hospitalized in the clinic?  *Please rate the following issues on a scale from 1 = strongly disagree to 10 = strongly agree.*   \|  \| 1 \| 2 \| 3 \| 4 \| 5 \| 6 \| 7 \| 8 \| 9 \| 10 \| \| --- \| --- \| --- \| --- \| --- \| --- \| --- \| --- \| --- \| --- \| --- \| \| ‘Nice change’ to the daily administration of antithyroid medication. \| o \| o \| o \| o \| o \| o \| o \| o \| o \| o \| \| Little difference to usual. \| o \| o \| o \| o \| o \| o \| o \| o \| o \| o \| \| I was really concerned. \| o \| o \| o \| o \| o \| o \| o \| o \| o \| o \| \| You/ other family members missed the cat a lot. \| o \| o \| o \| o \| o \| o \| o \| o \| o \| o \| \| The partner cat missed the cat a lot. \| o \| o \| o \| o \| o \| o \| o \| o \| o \| o \| \| I had the feeling, that my cat was in good care. \| o \| o \| o \| o \| o \| o \| o \| o \| o \| o \| | | | |
| 3. Was the reintegration of your cat at home difficult or troublesome?  *Please rate on a scale from 1= not a problem at all to 10= the cat couldn’t be reintegrated at all.*   \| 1 \| 2 \| 3 \| 4 \| 5 \| 6 \| 7 \| 8 \| 9 \| 10 \| \| --- \| --- \| --- \| --- \| --- \| --- \| --- \| --- \| --- \| --- \| \| o \| o \| o \| o \| o \| o \| o \| o \| o \| o \| | | | |
| 4. Radiation safety measures.  *Please rate the following issues on a scale from 1 = not a burden for me at all to 10 = extreme burden.*   \|  \| 1 \| 2 \| 3 \| 4 \| 5 \| 6 \| 7 \| 8 \| 9 \| 10 \| \| --- \| --- \| --- \| --- \| --- \| --- \| --- \| --- \| --- \| --- \| --- \| \| Was it a burden for your cat to abide by the radiation safety measures? \| o \| o \| o \| o \| o \| o \| o \| o \| o \| o \| \| Was it a burden for you to abide by the radiation safety measures? \| o \| o \| o \| o \| o \| o \| o \| o \| o \| o \| | | | |
| 5. How burdened were you by the following radiation safety measures?  *Please rate the following issues on a scale from 1 = not a burden for me at all to 10 = extreme burden.*   \|  \| 1 \| 2 \| 3 \| 4 \| 5 \| 6 \| 7 \| 8 \| 9 \| 10 \| \| --- \| --- \| --- \| --- \| --- \| --- \| --- \| --- \| --- \| --- \| --- \| \| To keep your cat indoors for 4 weeks. \| o \| o \| o \| o \| o \| o \| o \| o \| o \| o \| \| Having less than 2 hours close contact to your cat per day. \| o \| o \| o \| o \| o \| o \| o \| o \| o \| o \| \| That the cat litter needs to be stored separately. \| o \| o \| o \| o \| o \| o \| o \| o \| o \| o \| \| The effort to keep your cat separated. \| o \| o \| o \| o \| o \| o \| o \| o \| o \| o \| | | | |
| 6. Has the daily life improved after radioiodine treatment?  *Please rate on a scale from 1 = no change at all to 10 = extreme improvement.*   \| 1 \| 2 \| 3 \| 4 \| 5 \| 6 \| 7 \| 8 \| 9 \| 10 \| \| --- \| --- \| --- \| --- \| --- \| --- \| --- \| --- \| --- \| --- \| \| o \| o \| o \| o \| o \| o \| o \| o \| o \| o \| | | | |
| 7. When have you seen significant improvement of your cats’ symptoms? | | | - During the first month after radioiodine treatment - 1 to 2 months after radioiodine treatment - 3 to 6 months after radioiodine treatment - There has been no improvement. - The symptoms have worsened.   Other: _________________________________ |
| 8. How satisfied were you with the support provided by the radioiodine team of our clinic after the radioiodine treatment (questions, checkups, …)?  *Please rate on a scale from 1= not satisfied at all to 10= extremely satisfied.*   \| 1 \| 2 \| 3 \| 4 \| 5 \| 6 \| 7 \| 8 \| 9 \| 10 \| \| --- \| --- \| --- \| --- \| --- \| --- \| --- \| --- \| --- \| --- \| \| o \| o \| o \| o \| o \| o \| o \| o \| o \| o \| | | | |
| 9. What do you think about the regular veterinary re-examinations after the radioiodine treatment?  *Please rate on a scale from 1= does not apply at all to 10= applies extremely.*   \|  \| 1 \| 2 \| 3 \| 4 \| 5 \| 6 \| 7 \| 8 \| 9 \| 10 \| \| --- \| --- \| --- \| --- \| --- \| --- \| --- \| --- \| --- \| --- \| --- \| \| The re-examinations are too frequent. \| o \| o \| o \| o \| o \| o \| o \| o \| o \| o \| \| I would like to have more frequent re-examinations. \| o \| o \| o \| o \| o \| o \| o \| o \| o \| o \| \| I think the prescribed re-examinations are reasonable. \| o \| o \| o \| o \| o \| o \| o \| o \| o \| o \| \| The re-examinations put me under stress. \| o \| o \| o \| o \| o \| o \| o \| o \| o \| o \| \| The re-examinations put my cat under stress. \| o \| o \| o \| o \| o \| o \| o \| o \| o \| o \| \| The re-examinations are a financial burden for me. \| o \| o \| o \| o \| o \| o \| o \| o \| o \| o \| \| I think the re-examinations are unnecessary. \| o \| o \| o \| o \| o \| o \| o \| o \| o \| o \| | | | |
| 9. What do you think about the regular veterinary re-examinations after the radioiodine treatment?  *Please rate on a scale from 1= does not apply at all to 10= applies extremely.*   \|  \| 1 \| 2 \| 3 \| 4 \| 5 \| 6 \| 7 \| 8 \| 9 \| 10 \| \| --- \| --- \| --- \| --- \| --- \| --- \| --- \| --- \| --- \| --- \| --- \| \| The re-examinations are too frequent. \| o \| o \| o \| o \| o \| o \| o \| o \| o \| o \| \| I would like to have more frequent re-examinations. \| o \| o \| o \| o \| o \| o \| o \| o \| o \| o \| \| I think the prescribed re-examinations are reasonable. \| o \| o \| o \| o \| o \| o \| o \| o \| o \| o \| \| The re-examinations put me under stress. \| o \| o \| o \| o \| o \| o \| o \| o \| o \| o \| \| The re-examinations put my cat under stress. \| o \| o \| o \| o \| o \| o \| o \| o \| o \| o \| \| The re-examinations are a financial burden for me. \| o \| o \| o \| o \| o \| o \| o \| o \| o \| o \| \| I think the re-examinations are unnecessary. \| o \| o \| o \| o \| o \| o \| o \| o \| o \| o \| | | | |
| 10. Satisfaction  *Please rate the following issues on a scale from 1 = not content at all to 10 = extremely content.*   \|  \| 1 \| 2 \| 3 \| 4 \| 5 \| 6 \| 7 \| 8 \| 9 \| 10 \| \| --- \| --- \| --- \| --- \| --- \| --- \| --- \| --- \| --- \| --- \| --- \| \| How satisfied are you with your decision for your cat to undergo radioiodine treatment? \| o \| o \| o \| o \| o \| o \| o \| o \| o \| o \| \| How satisfied are you with the outcome of radioiodine treatment? \| o \| o \| o \| o \| o \| o \| o \| o \| o \| o \| | | | |
| 11. Would you recommend radioiodine treatment? | o Yes  o No  o I don’t know  o Other | | |
| 12. What are the main advantages of radioiodine treatment for you and your cat?  Please rate the following issues on a scale from 1 = strongly disagree at all to 10 = strongly agree.   \|  \| 1 \| 2 \| 3 \| 4 \| 5 \| 6 \| 7 \| 8 \| 9 \| 10 \| \| --- \| --- \| --- \| --- \| --- \| --- \| --- \| --- \| --- \| --- \| --- \| \| No regular administration of antithyroid medication. \| o \| o \| o \| o \| o \| o \| o \| o \| o \| o \| \| Your cat doesn’t have to be checked so often by the vet. \| o \| o \| o \| o \| o \| o \| o \| o \| o \| o \| \| You are less concerned about your cat’s condition. \| o \| o \| o \| o \| o \| o \| o \| o \| o \| o \| \| Possibility for your cat to be cured. \| o \| o \| o \| o \| o \| o \| o \| o \| o \| o \| \| Your cat is in better health than before radioiodine treatment. \| o \| o \| o \| o \| o \| o \| o \| o \| o \| o \| | | | |
| 13. To what extent do you think the following issues are disadvantages of the radioiodine treatment?  Please rate the following issues on a scale from 1 = does not apply at all to 10 = applies extremely.   \|  \| 1 \| 2 \| 3 \| 4 \| 5 \| 6 \| 7 \| 8 \| 9 \| 10 \| \| --- \| --- \| --- \| --- \| --- \| --- \| --- \| --- \| --- \| --- \| --- \| \| The hospitalization after the radioiodine treatment. \| o \| o \| o \| o \| o \| o \| o \| o \| o \| o \| \| Having to keep the cat indoor for another 4 weeks after radioiodine treatment. \| o \| o \| o \| o \| o \| o \| o \| o \| o \| o \| \| The costs of the radioiodine treatment. \| o \| o \| o \| o \| o \| o \| o \| o \| o \| o \| \| Having to keep your cat at a distance for another 4 weeks after radioiodine treatment. \| o \| o \| o \| o \| o \| o \| o \| o \| o \| o \| \| The long distance to the treatment facility in Giessen (Germany). \| o \| o \| o \| o \| o \| o \| o \| o \| o \| o \| \| Long waiting period for an appointment. \| o \| o \| o \| o \| o \| o \| o \| o \| o \| o \| \| Because my cat developed hypothyroidism and increased kidney values, I had to restart giving oral medications. \| o \| o \| o \| o \| o \| o \| o \| o \| o \| o \| \| The possible health risk for you and your family due to the residual radiation (e.g. when handling your cat or cleaning the litter box). \| o \| o \| o \| o \| o \| o \| o \| o \| o \| o \| | | | |

1. Socio-demographic data about the owners (n=77)

| **Question** | **Choice of options** | **Number n** | **%** |
| --- | --- | --- | --- |
| Age | Up to 30 years | 8 | 10.4 |
|  | 31 to 40 years | 16 | 20.8 |
|  | 41 to 50 years | 22 | 28.6 |
|  | Over 50 years | 31 | 40.3 |
|  | Prefer not to say | 0 | 0 |
|  | No answer | 0 | 0 |
| Gender | Female | 60 | 77.9 |
|  | Male | 17 | 22.1 |
|  | No Answer | 0 | 0 |
| Current country of residence | Germany | 77 | 100 |
| Having children living in the same household (age of the youngest child) | No | 66 | 85.7 |
|  | Younger than 3 years | 2 | 2.6 |
|  | 3 to 6 years | 2 | 2.6 |
|  | 7 to 12 years | 0 | 0 |
|  | 13 to 18 years | 3 | 3.9 |
|  | Older than 18 years | 4 | 5.2 |
|  | Prefer not to say | 0 | 0 |
|  | Other | 0 | 0 |
| Highest level of education | Doctorate (PhD) | 4 | 5.2 |
|  | Diploma | 4 | 5.2 |
|  | State examination | 8 | 10.4 |
|  | Master's degree | 12 | 15.6 |
|  | Bachelor's degree | 9 | 11.7 |
|  | Secondary education | 21 | 27.3 |
|  | Abitur or equivalent qualification | 10 | 13 |
|  | Realschule or equivalent qualification | 6 | 7.8 |
|  | Hauptschule or equivalent qualification | 2 | 2.6 |
|  | Prefer not to say | 1 | 1.3 |
|  | Other | 0 | 0 |
| Current work situation | Employed full-time | 35 | 45.5 |
|  | Employed part-time | 9 | 11.7 |
|  | Employed full-time (work from home) | 3 | 3.9 |
|  | Employed full-time (hybrid) | 13 | 10.4 |
|  | Unemployed | 2 | 16.9 |
|  | Retired | 9 | 11.7 |
|  | Unable to work | 1 | 1.3 |
|  | Prefer not to say | 2 | 2.6 |
| Who cares mainly for the cat? | You | 64 | 83.1 |
|  | Your partner | 1 | 1.3 |
|  | You and your partner | 9 | 11.7 |
|  | The whole family | 3 | 3.9 |
| Time spend together with the cat per day | More than 7 hours per day | 33 | 42.9 |
|  | Less than 7 hours per day | 44 | 57.1 |
| Is this cat the first cat | Yes | 17 | 22.1 |
|  | No | 60 | 77.9 |
| Owned a cat with hyperthyroidism before | Yes | 7 | 9.1 |
|  | No | 70 | 90.9 |
| How many cats are owned | One cat | 30 | 38.9 |
